# Hooked and Cooked: A Fish Killer Genome Exposed

**DOI:** 10.1371/journal.pgen.1003590

**Published:** 2013-06-13

**Authors:** Mark J. Banfield, Sophien Kamoun

**Affiliations:** 1 Department of Biological Chemistry, John Innes Centre, Norwich Research Park, Norwich, United Kingdom; 2 The Sainsbury Laboratory, Norwich Research Park, Norwich, United Kingdom; Virginia Tech, United States of America

Few microorganisms match the impact that the oomycetes have had on mankind. This distinct lineage of eukaryotes is well-known for its most notorious member, *Phytophthora infestans*, the agent of the nineteenth century Irish potato famine, and several other devastating pathogens of cultivated and wild plants [1]. Indeed, more than 60% of oomycete species infect plants [2]. Less known is the fact that many oomycetes are parasitic on animals, from freshwater fish and crustaceans to mammals, such as livestock, pets, and humans [3]. Animal parasitic oomycetes have received much less attention than their plant pathogenic kin, and our understanding of their virulence mechanisms is rudimentary. However, research momentum is poised to accelerate with the first report of the genome of an animal parasitic oomycete. In this issue of *PLOS Genetics*, Jiang *et al.* [4] describe the 63 Mbp genome sequence of the fish pathogen *Saprolegnia parasitica* and highlight a distinct repertoire of candidate virulence genes.

Members of the genus *Saprolegnia* cause the disease saprolegniosis in both farmed and wild freshwater fish, such as the northern pike (*Esox lucius*) [3], [5], [6]. The disease is distinguished by mycelial growth on the fish skin and fins that can be followed by fatal invasion of the pathogen into muscles and blood vessels. *Saprolegnia* is particularly destructive in aquaculture, an industry of growing importance given the increased global consumption of fish and the decline of wild fish stocks. In high-value salmon farms, *Saprolegnia* causes significant damage with loss of about 10% of hatched fish [6]. The problem was exacerbated by the worldwide ban on the organic dye malachite green, which was widely used for chemical control but, as a toxic carcinogen, poses a health hazard to consumers [6], [7]. Consequently, saprolegniosis emerged as a significant problem for the aquaculture industry, and a proposed ban on another disinfectant, formalin, will further compound the problem.

The genome sequenced by Jiang *et al.* is of *S. parasitica* strain CBS223.65, which was isolated from infected pike fish (*Esox lucius*). The compact 63 Mbp genome encodes 17,065 predicted gene models. At one gene per 2.6 kb, it is the most gene-dense oomycete genome sequenced to date [8]. Extensive regions of the genome display loss of heterozygosity, which has also been observed in other oomycete genomes [9]. This may be a driver of genetic diversity. However, despite some similarities, the *S. parasitica* genome turns out to be quite different from other sequenced oomycetes with both loss and gain/expansion of gene classes that are consistent with the pathogen's lifestyle. The genome displays a large complement of kinases (kinome), larger than that of humans, and an expanded set of enzymes involved in chitin metabolism. However, it is the apparent adaptation to animal parasitism that is particularly exciting.

Pathogens and their hosts are engaged in a constant molecular arms race, with pathogens deploying virulence proteins as weapons to subvert hosts for their own benefit. Plant pathogenic oomycetes secrete a large array of cell wall–degrading enzymes that act to break down this protective physical barrier. These enzymes are essentially absent in *S. parasitica* as animal cells lack a cell wall. In contrast, the secretome of *S. parasitica* is dominated by proteases and lectins. One protease, which is highly expressed in mycelia (SPRG_14567), specifically degrades trout immunoglobulin M (IgM). This may represent a virulence activity designed to evade pathogen recognition by the fish immune system, suggesting secreted proteases can act to suppress host defences and are not necessarily deployed to attack host tissue.

It is well established that many plant parasitic oomycete genomes encode expanded families of putative host-translocated effectors characterised by amino acid sequence motifs such as RXLR, LFLAK, and CHXC [10]. Many of these effectors are expected to manipulate plant immunity. Interestingly, these types of effectors are completely absent in *S. parasitica*. Perhaps the lack of these classes of host-translocated effectors in the genome suggests they are not important for *S. parasitica* pathogenesis? Perhaps these types of effectors are not well suited for supporting infection of fish cells? However, one protein, SpHtp1, has previously been shown to translocate into fish cells [11]. The degree to which *S. parasitica* relies on other effectors in addition to SpHtp1 will be an interesting question to address in the future.

**Figure 1:**
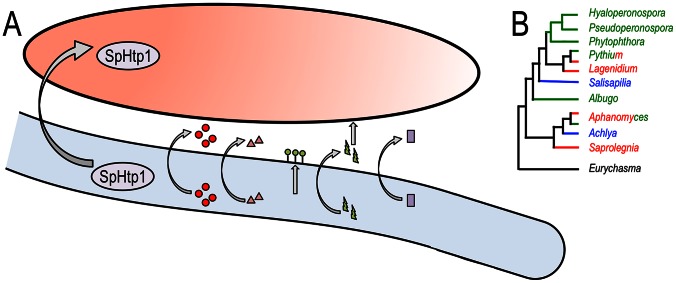
Multiple virulence factors are deployed by *Saprolegnia parasitica* and an overview of oomycete phylogeny. (A) Schematic representation of a *Saprolegnia parasitica* hypha (light blue) deploying virulence factors against a fish cell (salmon color). SpHtp1 is translocated inside the host cell, and other factors are secreted to the cell surface (lectins [green circles]) or the extracellular space (proteases [red circles], CHAPs [pink triangles], toxins [HlyE, which presumably targets the host membrane, green bolts] and nucleases [purple squares]). B. An overview of oomycete phylogeny. The main genera are displayed with the plant pathogenic lineages in green, animal parasites in red, and saprophytes in blue. Some genera, such as *Pythium* and *Aphanomyces*, include both plant and animal parasitic species. The early branching *Eurychasma* is an obligate pathogen of marine brown algae.

What other virulence proteins are deployed by *S. parasitica*? Remarkably, Jiang *et al.* discovered a large number of genes that are typical of animal parasites and are missing in plant pathogenic oomycetes. These include toxins (Haemolysin E-like), lectins and disintegrins (which may mediate host cell binding), CHAPs (cysteine, histidine-dependent amidohydrolases/peptidases), and secreted nucleases (Figure 1A). Even more remarkably, Jiang *et al.* show that these genes may have been acquired by horizontal gene transfer (HGT), possibly from bacteria. Further, the most abundant transposon in the *S. parasitica* genome belongs to the LINE repeat group. This repetitive element is prevalent in animal genomes and may have been acquired from an animal host. The role of the virulence factors acquired by HGT in progression of saprolegniosis will surely be a target for further study.

As highlighted by Jiang *et al.*, the genetic makeup of the fish parasite *S. parasitica* turned out to be markedly different from those of plant pathogenic oomycetes. Thus, it is important to sample across a diverse range of parasitic and saprophytic lifestyles to gain a thorough understanding of the structure and function of oomycete genomes (an overview of oomycete phylogeny is given in Figure 1B). To date, genome sequence analyses of plant pathogenic oomycetes of the genera *Albugo*, *Hyaloperonsopora*, *Phytophthora*, *Pseudoperonospora*, and *Pythium* have been published [9], [12]–[19]. Yet we still await the complete genome sequence of saprophytic species and additional animal parasites such as *Aphanomyces* spp. [20]. With these genome sequences in hand, comparative analyses will shed further light on how these enigmatic eukaryotes have adapted to a variety of ecological niches and host species.
